# Supplementary material for: Nanogenerator-based dual-functional and self-powered thin patch loudspeaker or microphone for flexible electronics
Source: Nat Commun. 2017 May 16;8:15310. doi: 10.1038/ncomms15310 (PMC5440853; doi:10.1038/ncomms15310)
Supplement: Supplementary Information — Supplementary Figures and Supplementary Notes [file ncomms15310-s1.pdf]

## Supplementary Figures

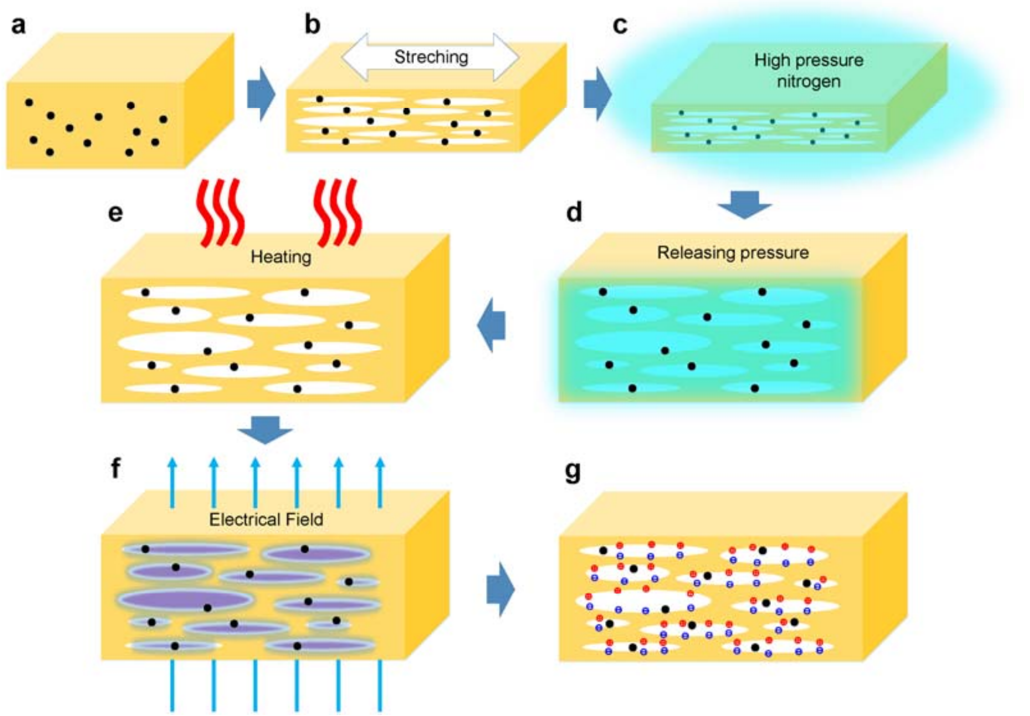


**Supplementary Figure 1.** Schematic illustration of the fabrication process of polypropylene ferroelectric film. (a) Polypropylene film is filled with silicate particles. (b) Film is under stretching. (c) Film is in high pressure (5 MPa) nitrogen environment, and nitrogen eventually diffuse into the internal voids. (d) Suddenly release the high pressure of nitrogen, results in the swell of the internal voids. (e) Film experience 100°C heat treatment. (f) Microplasma discharging inside the voids by applying high electric field to ionize the gas molecules. (g) Positive charge and negative charge separated and located at the surfaces of the voids after discharge, forming the giant dipoles.

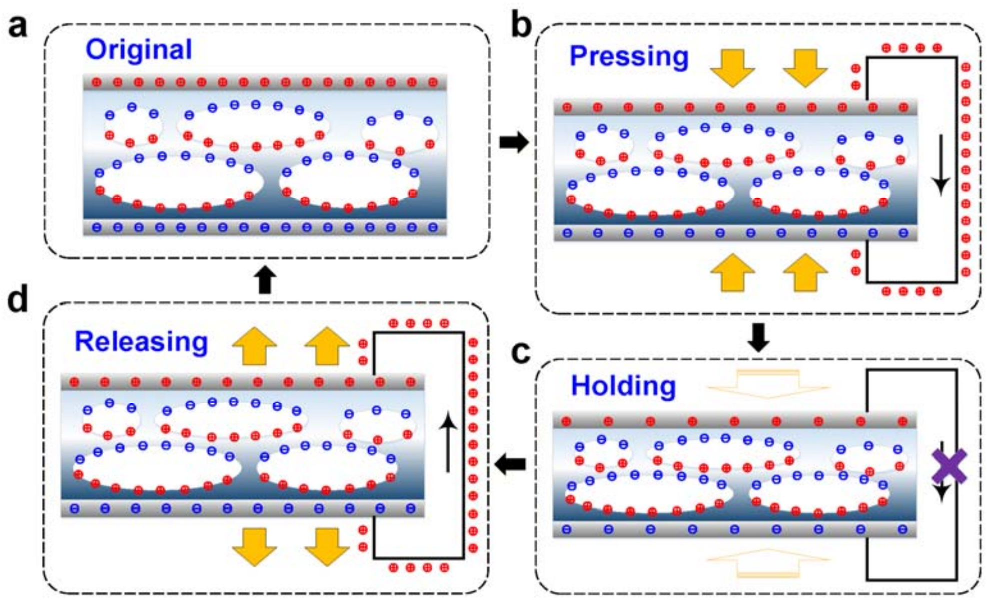


**Supplementary Figure 2.** Detailed illustration of direct electromechanical interaction effect of FENG with the flow of charges under external pressure in the thickness direction. (a) Original state without external pressure. (b) Pressing in thickness direction, resulting in the clockwise flow of positive charges. (c) Holding the same pressure produces no charge flow. (d) Releasing allows the positive charges flow counterclockwise, restoring to original state.

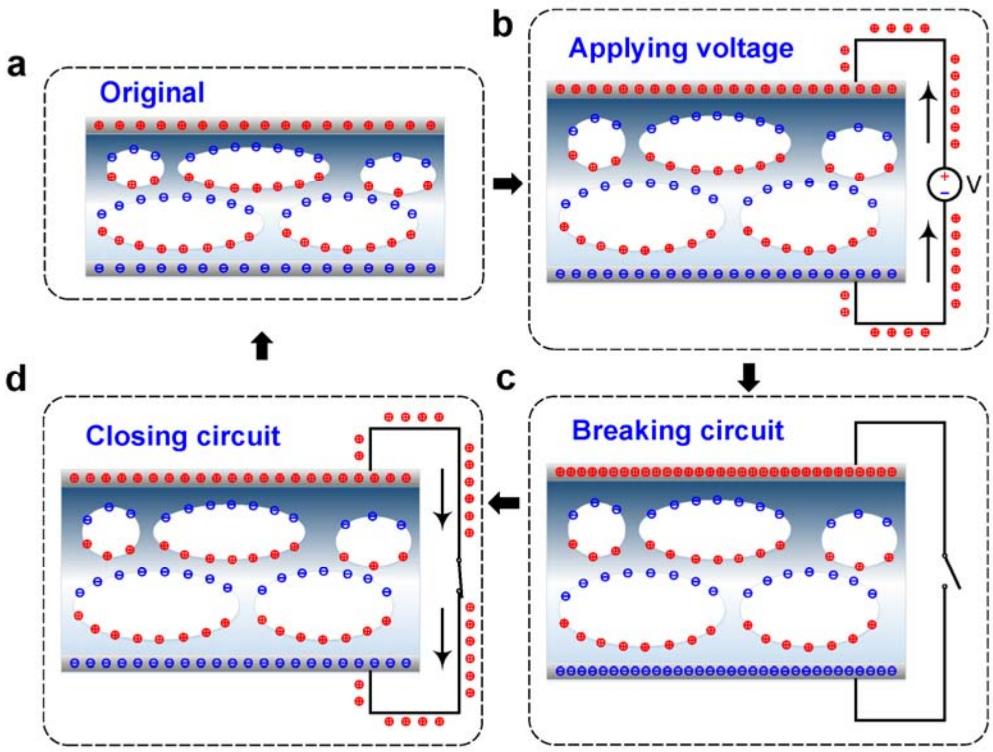


**Supplementary Figure 3.** Detailed illustration of reverse electromechanical interaction effect of FENG with the flow of charges under external potential. (a) Original state without voltage applied. (b) Applying voltage to the electrodes, resulting in counterclockwise flow of positive charges, and the increase of charge density on the electrodes, which in turn increase the thickness of FENG. (c) Breaking the circuit prevents further charge accumulation and thickness increase. (d) Closing the circuit allows the charges to flow back clockwise, restoring the thickness to original state.

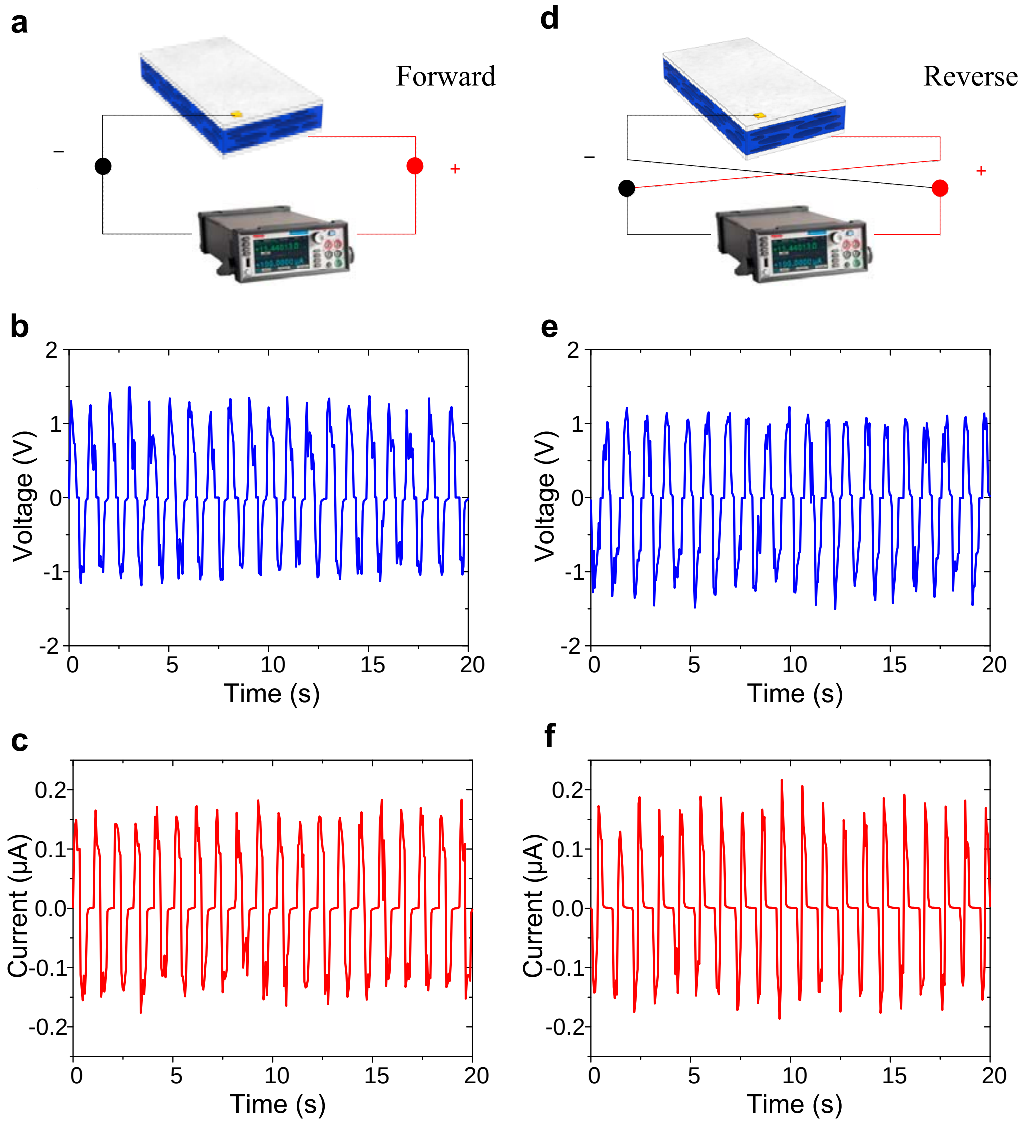


**Supplementary Figure 4.** Output electrical signal of FENG with area of 4 $cm^2$ under mechanical loading test. (a-c) Measured results with electrodes of FENG are connected forward. (a) Forward connection. (b) Open-circuit voltage. (c) Short-circuit current. (d-f) Measured results with electrodes of FENG connected in reverse configuration. (d) Reverse connection. (e) Open-circuit voltage. (f) Short-circuit current.

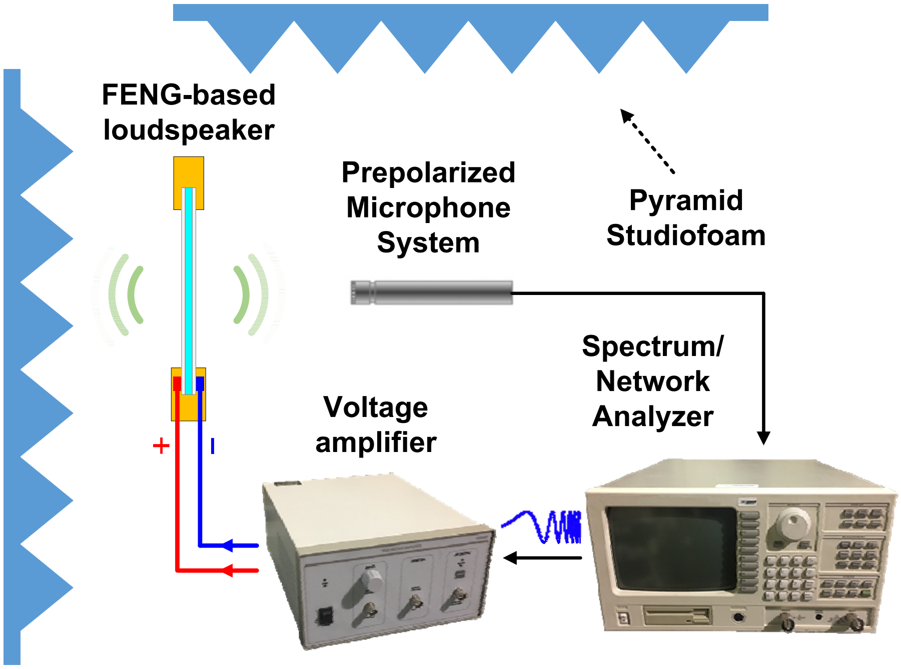


**Supplementary Figure 5.** Measurement and connection scheme of SPL amplitude-frequency response for FENG-based loudspeaker.

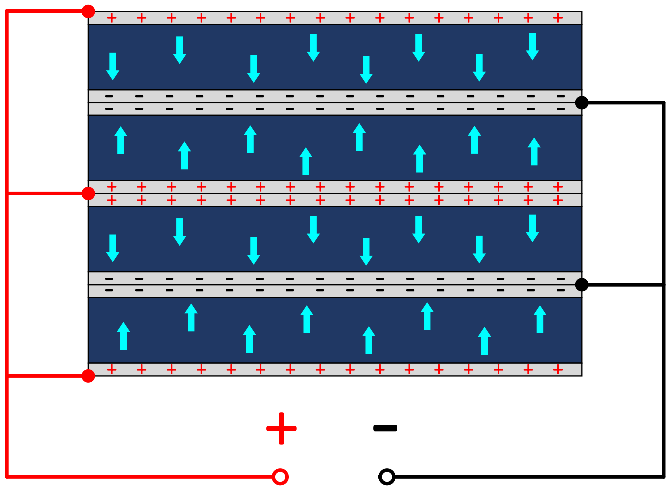

**Supplementary Figure 6.** Schematic illustration of the stacked multi-layers structure of FENG-based acoustic device.

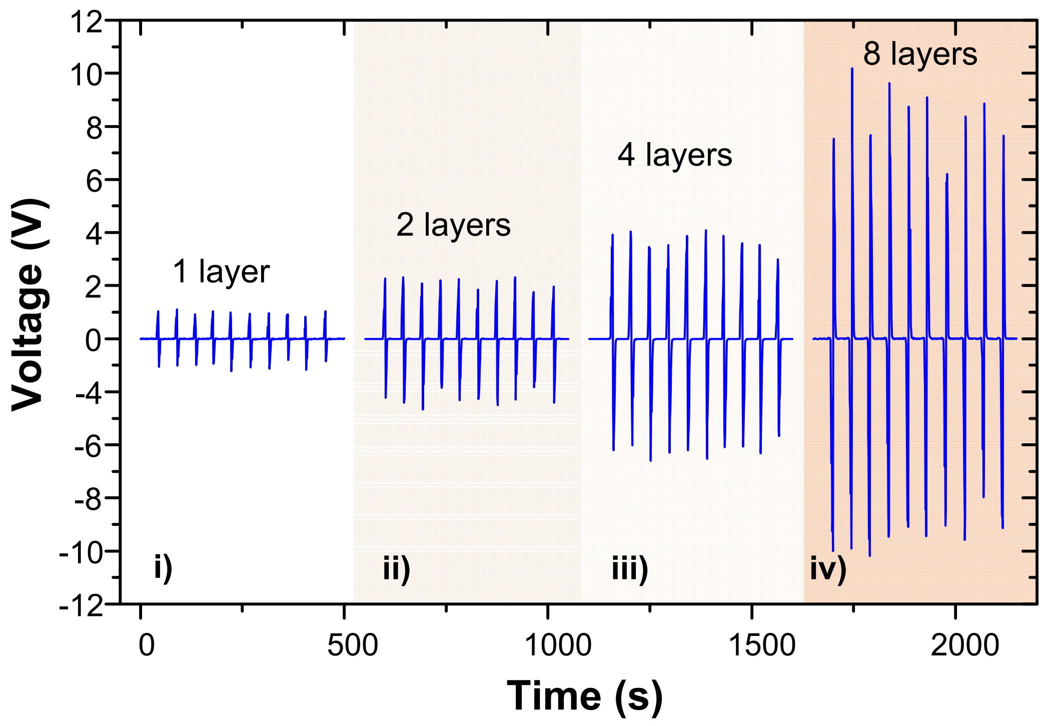


**Supplementary Figure 7.** Comparison of voltage generated by FENG under periodic pressing between i) one layer, ii) two layers, iii) three layers and iv) four layers.

# Supplementary Notes

## Supplementary Note 1: FENG-based music playing flag

A tablet was used to play the MSU fight song. The signal from the tablet was sent to an audio amplifier. After passing through a voltage amplifier, the electrical signal transformed into sound wave by means of the developed FENG-based music play flag. The music includes different marching band instruments, which can be recognized with high fidelity. The flag is flexible, and the video shows the flag waving due to wind while playing music.

## Supplementary Note 2: FENG-based sound recording

A commercial available loudspeaker plays the symphony from the tablet. Meanwhile, the FENG-based thin film microphone patch was recording the sound wave generated from the commercial loudspeaker. The acquired signal from the FENG-based microphone was sent to the audio card of a personal computer. After the recording process was done, we used the personal computer to broadcast the symphony which was just recorded in order to check out its fidelity. By comparing the original music and the recorded music, it can be seen that our developed FENG-based thin film sound recording technology successfully restored the original music.

## Supplementary Note 3: FENG-based identity recognition for privacy security application

To demonstrate the potential privacy security application for flexible electronic device based on the FENG, we used a log-in interface of personal computer to recognize the identities of three users who request for access. The personal computer is belong to administrator (third user in the video), so only he has the access to it. Rather than typing in keywords to log in, this computer allows the users to speak voice code to request for access, which is convenient and necessary for protecting the personal information stored in wearable/portable devices. For the unauthorized users #1 and #2, even though they obtained the correct voice code, when they spoke the voice code for the purpose of logging in, the system recognized their identities by using the FENG-based microphone and analyzing their voice information. Because their voiceprints information didn’t match the database, the computer declined their request for access. Access is only granted when the administrator speaks the right voice code; thus enabling two security layers: password and voice.
